# Nervous Diseases

**Published:** 1905-11-11

**Authors:** 


### PROGRESS IN MEDICINE AND SURGERY.

### NERVOUS DISEASES.

(*Continued from page* 87.)

**Tabes Dorsalis.**—The nature of the pathological changes found in tabes and their causes have received much consideration in modern times, and Gowers [9] has discussed the subject in a recent lecture, in which, however, he deprecates the idea of having said anything new. But if this be so, he has at least presented recognised ideas upon the subject in a fresh and vivid form. Of the many possible symptoms present in the disease he takes two—loss of the knee jerk and incoordination of the legs, as essential in a case deserving the name of locomotor ataxy. To these may be added another, less readily recognised—diminution of muscular tone, "hypotonus" or "undertone." The existence normally of a certain degree of tonic muscular contraction is shown by the slight continuous resistance felt on making a passive movement. In tabes this is lessened or lost. It depends on impairment of the peripheral sensory nerves of the muscle—an impairment shown also by the insensibility of the tabetic to muscular pain produced by a strong faradic contraction. This change in the afferent muscle nerves explains also the loss of the knee jerk, for which the word "tendon reflex" is a misnomer. The tap on the tendon merely excites the local muscular contraction due really to the muscle tone and dependent on the spinal cord. Tone is essentially a muscle reflex process. The loss of the knee jerk is a delicate indication of the impairment of the afferent muscle nerves, shown later by the loss of muscular sensibility. This spinal reflex process must undoubtedly play an important part in the adjustment of muscular contractions and in determining their relations for the movements arranged in the cerebral centres. Irregularity, delay, or feebleness in the transmission of impulses must derange this spinal mechanism, and be one cause of the ataxy present in tabes. Another element is probably dependent on the degeneration of the posterior median columns, which are made up of the muscle neurons and transmit muscle impulses to the medulla, thence probably to the cerebellum, where they are coordinated with others to guide the motor cortex. The ataxy and the lost knee jerk of tabes may therefore be entirely due to the degeneration of the sensory muscle neurons and to the effect of the loss of their impulses on the spinal, cerebellar, and cerebral processes. Although usually associated with failure of cutaneous sensibility and defect of afferent impulses from the joints and tendons, these are of less importance than the failure of muscular sensibility. In pure tabes the motor cells and fibres are not affected. The essential changes are in the neurons which have their vital centres in the posterior spinal ganglia—that is, in the posterior nerve roots and the median dorsal columns on the one side and on the sensory nerves on the other. It is natural, then, to look for changes in the fountain-head of these neurons—the cells of the posterior ganglia themselves. Yet microscopic and chemical examination has in many cases, though not in all, found them apparently normal. How, then, can a failure in their vitality be the cause of the essential symptoms of tabes? In reply, two important points must be remembered—the first, that molecular changes must attain a relatively vast degree to be visible, and changes far too minute to be appreciated may cause grave impairment of function; the second, that the greater the distance of the fibre from its vital centre (the cell), the less its vital power and the less its ability to resist morbid agents. Hence, as in every peripheral neuritis, the distal portion may degenerate long before the cell body shows visible change. Failure to appreciate change, therefore, in the ganglia does not necessarily mean absence of change. The cause of the degeneration is next considered by Gowers. It is an acquired degeneration—selective, bilateral; it must therefore be due to a toxin circulating in the blood, the action of which is analogous to that of arsenic and alcohol. Each of these poisons, indeed, occasionally selects the sensory neurons, and the lost knee jerk and incoordination of tabes are thus simulated, although usually the motor neurons are chiefly affected. Probably the selection of one or the other depends on certain special changes of the nutritional plasma, of which we have no other evidence, the differences being so minute as to defy perception. Similarly the toxins of the acute specific fevers, as in diphtheria, or the disordered chemical reactions constituting diabetes, may exceptionally give rise to the symptoms and morbid anatomy of tabes. The toxic nature of tabes having been demonstrated, its relation to syphilis is next discussed. Evidence of the association with the latter is purely that of sequence, but it is very strong. In 100 consecutive private male cases Gowers obtained a history of a chancre in 80, and a history of gonorrhœa incurred or risked in the remainder. Erb's much larger numbers are even more significant, the primary sore having been present in 89½ per cent. It is of much importance to note that in 124 cases of undoubted syphilitic skin disease investigated by Pernet for purposes of comparison, a history of chancre was obtained in exactly the same proportion—80 per cent. The disease occasionally develops also in the wife of a tabetic who has had syphilis before his marriage, and inherited syphilis has been present in all the few reported cases of "juvenile tabes." One great difficulty in connection with syphilis must be faced. If tabes is due to a toxin, and that toxin is produced by the supposed organism of syphilis—which has not yet been identified, although the recent investigations by Metchnikoff and Roux [10] on the spirochaete pallida of Schaudinn are promising—the toxin in this case must act not during the active stage of the malady, as is usual, but when all other active symptoms have long since subsided. The theory that the toxin acts merely by producing lessened vital resistance is not tenable in view of the remarkable degree of recovery which is occasionally observed. The facts seem best explained by introducing another unknown agent, an idea expressed thus by Neisser: Tabes is the result of a syphilitic toxin plus *x*, *x* being the unknown intensifying influence. Probably processes once set going in the complex laboratory of the body persist and increase. That such changes, apparently trifling, do persist and remain potent is shown, *e.g.*, by the life-long immunity permanently secured by an acute specific infection such as scarlet fever. A perverted process may conceivably become greater by constant action, and thus syphilis may cause changes in chemical processes which result, after years, in the generation of a nerve poison. Such action may cooperate from time to time with other morbid influences, or be assisted by general lowered vitality or local over-exertion, and in this way the development of the disease, its renewed outbursts of activity or partial recoveries, may be explained. Lesser [11] adduces post-mortem evidence in favour of the connection of syphilis with tabes. He found traces of syphilis post-mortem in 28 per cent. of tabetic patients, and only in 9.5 per cent. of general patients over the age of thirty-five. He regards tabes as a "quartary" syphilitic manifestation, due to parenchymatous degeneration of the posterior columns of the cord secondary to an overgrowth of interstitial, neuroglial, tissue. The interstitial overgrowth constitutes the quartary syphilitic lesion which, in his view, is exactly comparable to that which occurs in syphilitic fibrosis of the liver, which is selective, produces a secondary cell degeneration, and is uninfluenced by anti-syphilitic remedies. It will be seen that this view neglects the condition of the posterior ganglia, regarded by Gowers as in all probability at fault. Lesser also points to the frequent association of tabes with aneurysm, also frequently an interstitial syphilitic manifestation. He has noted the coincidence in every fifth patient. A case of locomotor ataxy associated with fine rhythmical tremor, such as occurs in paralysis agitans, has been observed by Rhein.[12] Hindsdall [13] has found good results from early treatment by rest, massage, and electricity. Bradshaw [14] points to the value of permanent acceleration of the pulse in the early diagnosis of tabes.

[9] Brit. Med. Jour., July 8. [10] Ibid., May 27, p. 1178. [11] Jour. Nerv. and Ment. Dis., March, p. 205. [12] Ibid., April, p. 275. [13] Ibid., p. 273. [14] Brit. Med. Jour., July 8, p. 61.

